# Supplementary material for: Development and preliminary validation of infrared spectroscopic device for transdermal assessment of elevated cardiac troponin
Source: Commun Med (Lond). 2022 Apr 13;2:42. doi: 10.1038/s43856-022-00104-9 (PMC9053220; doi:10.1038/s43856-022-00104-9)
Supplement: Supplementary file 2 — Reporting Summary [file 43856_2022_104_MOESM2_ESM.pdf]

nature research

Corresponding author(s): Partho P. Sengupta

Last updated by author(s): Mar 15, 2022

Reporting Summary

Nature Research wishes to improve the reproducibility of the work that we publish. This form provides structure for consistency and transparency in reporting. For further information on Nature Research policies, see our Editorial Policies and the Editorial Policy Checklist.

Statistics

For all statistical analyses, confirm that the following items are present in the figure legend, table legend, main text, or Methods section.

n/a	Confirmed	
☐	☒	The exact sample size (n) for each experimental group/condition, given as a discrete number and unit of measurement
☐	☒	A statement on whether measurements were taken from distinct samples or whether the same sample was measured repeatedly
☐	☒	The statistical test(s) used AND whether they are one- or two-sided<br>*Only common tests should be described solely by name; describe more complex techniques in the Methods section.*
☐	☒	A description of all covariates tested
☐	☒	A description of any assumptions or corrections, such as tests of normality and adjustment for multiple comparisons
☐	☒	A full description of the statistical parameters including central tendency (e.g. means) or other basic estimates (e.g. regression coefficient) AND variation (e.g. standard deviation) or associated estimates of uncertainty (e.g. confidence intervals)
☐	☒	For null hypothesis testing, the test statistic (e.g. F, t, r) with confidence intervals, effect sizes, degrees of freedom and P value noted<br>*Give P values as exact values whenever suitable.*
☒	☐	For Bayesian analysis, information on the choice of priors and Markov chain Monte Carlo settings
☒	☐	For hierarchical and complex designs, identification of the appropriate level for tests and full reporting of outcomes
☐	☒	Estimates of effect sizes (e.g. Cohen's d, Pearson's r), indicating how they were calculated

Our web collection on statistics for biologists contains articles on many of the points above.

Software and code

Policy information about availability of computer code

Data collection	We used Python 3.7.1 for our analysis. We have used NumPy 1.19. 2, SciPy 1.5.0, OPUS 7.2, scikit-learn 0.24.0 for different numerical calculation and statistical analysis.
Data analysis	We used Python 3.7.1 for our analysis. We have used NumPy 1.19. 2, SciPy 1.5.0, OPUS 7.2, scikit-learn 0.24.0 for different numerical calculation and statistical analysis.

For manuscripts utilizing custom algorithms or software that are central to the research but not yet described in published literature, software must be made available to editors and reviewers. We strongly encourage code deposition in a community repository (e.g. GitHub). See the Nature Research guidelines for submitting code & software for further information.

Data

Policy information about availability of data

All manuscripts must include a data availability statement. This statement should provide the following information, where applicable:

- Accession codes, unique identifiers, or web links for publicly available datasets
- A list of figures that have associated raw data
- A description of any restrictions on data availability

The datasets generated during and/or analyzed during the current study, including source data for the figures, are available in the Figshare platform at https://doi.org/10.6084/m9.figshare.c.5871056

Field-specific reporting

Please select the one below that is the best fit for your research. If you are not sure, read the appropriate sections before making your selection.

☒ Life sciences ☐ Behavioural & social sciences ☐ Ecological, evolutionary & environmental sciences

For a reference copy of the document with all sections, see nature.com/documents/nr-reporting-summary-flat.pdf

Life sciences study design

All studies must disclose on these points even when the disclosure is negative.

Sample size	Considering this was a pilot study, neither the effect size nor the power was not fully known. Hence a sample size calculation was not performed prior to the study.
Data exclusions	There were no data points excluded that have been presented as graphs in the article. It is has also been alluded to in the article what the sensitivity and specificity would be if some identified data points were to be excluded based on observed user error.
Replication	In the transdermal measurements, the measurement was repeated 10 times for each datapoint. As the system reaches equilibrium, the optical output changes before reaching equilibrium. To properly represent this, the first three repeats were excluded for all datapoints.
Randomization	The only inclusion criterion being patients under suspicion of Acute Coronary Syndrome (ACS), all other patient demographic and parameters are random.
Blinding	The data was blinded during data analysis

Reporting for specific materials, systems and methods

We require information from authors about some types of materials, experimental systems and methods used in many studies. Here, indicate whether each material, system or method listed is relevant to your study. If you are not sure if a list item applies to your research, read the appropriate section before selecting a response.

Materials & experimental systems

n/a	Involved in the study	
☒	☐	Antibodies
☒	☐	Eukaryotic cell lines
☒	☐	Palaeontology and archaeology
☒	☐	Animals and other organisms
☐	☒	Human research participants
☒	☐	Clinical data
☒	☐	Dual use research of concern

Methods

n/a	Involved in the study	
☒	☐	ChIP-seq
☒	☐	Flow cytometry
☒	☐	MRI-based neuroimaging

Human research participants

Policy information about studies involving human research participants

Population characteristics	Patients were recruited strictly based on the presentation of chest pain under suspicion of ACS. The following population characteristics were recorded: Patient number, Patient Demographics, Date and Time stamp of presentation, Diabetes, Hypertension, On dialysis (CKD, ESRD), Smoking/Tobacco Chewing, Hypercholestrolemia, Symptoms at admission, Presenting Clinical Sx/Differentials, Prior History of Cardiac Disease, Previous Stress Test (If available), Current CAG findings, Current CAG (Intervention), Current Echo (Ejection Fraction), Current Echo (Wall Motion Abnormalities), Current Stress Echo (Color Kinesis), "Wrist wearable measurement (RCE)Date & Time stamp", Lab measurements, Diagnosis Medications.
Recruitment	Patients were recruited based only on the inclusion criterion being, "patient under suspicion of ACS".
Ethics oversight	Study protocol was approved by the Sengupta Hospital and Research Institute IRB Ethics Committee and in full compliance with the Declaration of Helenski. The other study mentioned in the article involves retrospective analysis on de-identified blood samples for which IRB doesn't apply.

Note that full information on the approval of the study protocol must also be provided in the manuscript.
